# Mental health treatment in Kenya: task-sharing challenges and opportunities among informal health providers

**DOI:** 10.1186/s13033-017-0152-4

**Published:** 2017-08-01

**Authors:** Christine W. Musyimi, Victoria N. Mutiso, David M. Ndetei, Isabel Unanue, Dhru Desai, Sita G. Patel, Abednego M. Musau, David C. Henderson, Erick S. Nandoya, Joske Bunders

**Affiliations:** 1 Africa Mental Health Foundation, Mawensi Road, Off Elgon Road, Mawensi Garden, P.O Box 48423, Nairobi, 00100 Kenya; 2 Vrije Universiteit, De Boelelaan 1105, 1081 HV Amsterdam, Netherlands; 3 University of Nairobi, P.O Box 30197, Nairobi, 00100 Kenya; 4 Palo Alto University, 1791 Arastradero Road, Palo Alto, CA 94304 USA; 5 Boston University School of Medicine, 72 E. Concord St, Boston, MA 02118 USA; 6 Harvard Medical School, 25 Shattuck Street, Boston, MA 02115 USA

**Keywords:** Informal health providers, Task sharing, Mental health, Challenges, Kenya

## Abstract

**Background:**

The study was conducted to explore challenges faced by trained informal health providers referring individuals with suspected mental disorders for treatment, and potential opportunities to counter these challenges.

**Methods:**

The study used a qualitative focus group approach. It involved community health workers, traditional and faith healers from Makueni County in Kenya. Ten Focus Group Discussions were conducted in the local language, recorded and transcribed verbatim and translated. Using a thematic analysis approach, data were entered into NVivo 7 for analysis and coding.

**Results:**

Results demonstrate that during the initial intake phase, challenges included patients’ mistrust of informal health providers and cultural misunderstanding and stigma related to mental illness. Between initial intake and treatment, challenges related to resource barriers, resistance to treatment and limitations of the referral system. Treatment infrastructure issues were reported during the treatment phase. Various suggestions for solving these challenges were made at each phase.

**Conclusions:**

These findings illustrate the commitment of informal health providers who have limited training to a task-sharing model under difficult situations to increase patients’ access to mental health services and quality care. With the identified opportunities, the expansion of this type of research has promising implications for rural communities.

## Background

Despite the high prevalence of mental disorders in low- and middle-income countries (LMICs), international health surveys show that only 80% of those with serious mental disorders receive any treatment [1]. To address this gap, Lancet Global Mental Health Group [2] and World Health Organization [3] have recommended harnessing and fostering local human resources and creating collaborative, task-sharing models. Task-sharing refers to the delegation of tasks historically performed by mental health professionals (e.g., diagnoses, counselling, referral) to non-specialist health workers or informal health providers (IHPs) [4]. In LMICs, task-sharing models present various benefits. Firstly, the number of IHPs far surpasses the amount of mental health professionals available thus more people, including those living in remote rural areas, can be reached [4, 5]. Secondly, the tasks IHPs can undertake are extensive including psycho-education, monitoring drug adherence, treating common mental disorders, and detecting and referring individuals [3, 6]. Thirdly, research has already shown that task-sharing is effective; it has successfully improved outcomes for depression, PTSD, dementia, and alcohol use disorders [7].

Another important benefit is that IHPs are often more culturally-accepted and represent the first recourse of treatment in community settings [5]. The particular IHP groups included in this study—community health workers (CHWs), faith healers (FHs) and traditional healers (THs) represent three groups that are widely sought and culturally-embedded in the Kenyan context. Each of these groups have previously been trained and involved in task-sharing initiatives [8, 9]. In Kenya, CHWs volunteer and work collaboratively with primary health centres to detect, treat, and promote health in their respective health districts but only through recent initiatives have CHWs begun to address mental disorders [10, 11]. Traditional and faith healers, in contrast, are frequently used by community members for the treatment of mental disorders [12, 13]. These two groups, however, have historically worked in parallel, rather than in collaboration with, primary health centres and hospitals [14]. This is in large part because traditional and faith healers promote indigenous, non-allopathic healing methods such as consulting ancestral spirits which, although accepted by locals, are often shunned by biomedical practitioners [5, 14].

However, given traditional and faith healers’ broad cultural acceptance and presence, this trend is rapidly changing. In 2002, the World Health Organization [15] advocated for the inclusion of traditional healers in its Traditional Medicine Strategy. Many African countries have approved the Traditional Health Practitioners bill that recognizes and regulates the practice of traditional health practitioners. Traditional health practitioners in Kenya have mostly been known to use herbal medication, counselling and consulting ancestral spirits for the treatment of various illnesses [12]. Furthermore, the National Health Sector Strategic Plan II (NHSSP II) recognizes the role of CHWs to provide community-based services including awareness and strengthening linkages between the community and health facility [13, 16]. A growing body of research also demonstrates the widespread use of practitioners, and shows that integrating them into the treatment of mental health problems can lead to effective and culturally sensitive interventions [5]. In Ghana, for instance, Ae-Ngibise and colleagues [14] conducted a qualitative study with over 100 respondents including health providers, users of psychiatric services, policy makers, and traditional health practitioners such as traditional and faith healers. Respondents said that they sought traditional health practitioners because they provided accessible and affordable care, counselling, and shared similar cultural perceptions of mental disorders. In Kenya, Ndetei and colleagues [17] interviewed over 50 traditional healers and 300 patients and found similar results, with patients reporting healers to be more affordable, accessible and approachable than formal health professionals.

Additional qualitative research has set the groundwork for establishing partnerships between Western medical professionals and traditional health practitioners [5, 18–20]. In each case, research results have demonstrated that traditional health practitioners are open and willing to collaborate with formal medical officers but are stigmatized by the biomedical community practitioners [5, 18–20]. Although there have been suggestions to form a dialogue to reduce mistrust and resolve collaboration issues [20], prior research has revealed the failure of many projects to provide systematic follow-up to IHPs after their initial training, which means that barriers remain even after they assimilate the new knowledge and integrate it into their practice [21].

Similarly, task-sharing is not necessarily a solution for countries with limited mental health resources in that multiple elements have to be taken into account in order for this strategy to be effective [9]. Research from recent reviews has not yet specifically demonstrated how task-sharing with IHPs is effective in practice, the unique challenges and opportunities that emerge during collaboration, and how these may manifest in the Kenyan cultural context [4, 8]. Further exploration of challenges faced by these IHPs during collaboration and using task-sharing solutions to overcome these challenges is thus an important next step in research.

In Kenya, a collaborative framework that includes IHPs is well suited to address the realities of the country’s mental health care system. Similar to other African countries, Kenya has a shortage of mental health professionals. Many individuals, especially those living in rural areas, often seek IHPs such as THs and FHs to treat mental disorders [17]. Task-sharing with IHPs could be a powerful strategy to address the country’s mental health challenges. However, despite the recent systematic attempts to establish a formal dialogue between formal and informal health providers [20], little is known about the challenges that IHPs face as they become trained to function in a system that addresses mental health needs. To address this gap, this study used a qualitative focus group approach to explore the challenges faced by trained IHPs who refer individuals with mental disorders for conventional treatment and potential opportunities to reduce these challenges.

## Methods

The study used a qualitative approach that involved three sets of participants (THs, FHs and CHWs) from Makueni County in Kenya, who were selected to participate in a study on scaling up community mental health in Kenya using informal and formal health providers. The initial selection was based on registration by an association for THs, affiliation to a place of worship for FHs, affiliation to a health facility for CHWs and finally interest in participation in the study. Proximity to 20 health facilities was selected administratively in collaboration with the Makueni County health management team to represent levels two (dispensaries), three (health centres), four (sub-county hospitals) and five (County hospitals) facilities. The presence of electricity in the facilities due to remote data entry was another criterion used to select the IHPs in order to facilitate referral of patients. Although six to nine IHPs were selected for each facility, only a subset was used for this phase, determined by dividing the facilities into three clusters to facilitate geographical variability, then randomly selecting participants for the focus groups from each cluster, which resulted in at least three separate focus groups (FHs, THs and CHWs) per cluster.

Initially, a total of nine focus group discussions (FGDs) (three each for THs, FHs and CHWs) each consisting of between eight and ten participants were conducted in February 2016. In addition, a fourth FGD for CHWs was conducted to achieve saturation of all new information since it is impossible to predetermine the sample size for qualitative studies [22]. All participants had been trained 3 months before conducting the FGDs in a classroom setting by a mental health specialist on how to identify and refer patients with priority mental illnesses to the nearest health facilities. The training was conducted for 2 days on the types of priority mental disorders, their prevalence estimates, myths and misconceptions about mental illness, consenting procedures and how to identify the disorders using the mental health Global Action Programme Intervention Guide (mhGAP-IG) master chart which provides the common presentations for mental disorders [23]. This was crucial in order to ensure uniformity of terms and similar identification criteria for mental disorders. Basic information about mental illness was also included in the training since the participants did not have prior formal training on mental health, unlike non-specialists based in health facilities who have basic training in health care including some components of mental health care. Furthermore, demonstrations and role-plays were important components of the training to promote experiential learning.

Referral to health facilities for treatment by primary health care providers was based on achieving a certain threshold for a mental disorder as per symptom presentation in the mhGAP-IG master chart rather than clinical judgement, which may have eliminated culturally specific conceptualizations of mental ill-health found in many African Countries. Performance-based incentives were provided for the IHPs during screening and referral of patients. This initiative was performed in collaboration with the County government of Makueni to promote sustainability. Makueni County is the first to integrate nearly all categories of IHPs in mental health care as stated in the Kenya mental health policy 2015–2030. Moreover, this was an added advantage in terms of sustainability of the services for IHPs, since they acquired knowledge on identification of mental disorders for use during routine practice.

Before every FGD, all participants consented and confidentiality measures were explained and observed. All FGDs were carried out in the local language by facilitators with a minimum of a bachelor’s degree in a health-related field and at least 2 years’ experience in community mental health research, in a central private room depending on the location of the facilities, and took about 2 h. The focus groups explored participants’ views about challenges they faced during practice, including referral of patients with mental illness to formal health facilities. All FGDs were audio-recorded, transcribed verbatim and translated before analysis. In order to confirm the quality of the translations, the final versions were reviewed by an independent social scientist with a background in medical anthropology and fluent in the local language.

### Data analysis

All data were entered into NVivo software version 7 for analysis and coding. Using a thematic content analysis approach [24], the data were analysed in four stages, and included analyst triangulation [25]. First, two independent researchers reviewed all data for emergent themes and developed a thematic structure through an iterative process, followed by re-coding data into the emergent themes. All coding was discussed, any discrepancies resolved, and full agreement reached.

## Results

Group discussions illuminated a series of challenges that arose at distinct phases in the referral process. See Table 1 for a coding structure reflecting frequencies in challenge domains and related sub-themes. At the screening phase, when the IHPs first saw the patients, interviewees reported challenges such as mistrust towards them as health providers, as well as cultural misunderstanding and stigma related to mental illness. Between screening and treatment, challenges related to resource barriers (mentioned 61 times), resistance to treatment, and limitations of the referral system. Challenges mentioned during the treatment phase were related to treatment infrastructure issues and were mentioned 39 times.

**Table 1:** Frequencies within challenge domains and related sub-themes

	Definition	Frequency
Screening intake		
1. Mistrust of informal health providers	Patients tend to have negative biases towards healers	40
1.1 Belief system	Patients’ belief systems are in opposition to those of healers	14
1.2 Secondary gain	Patients assume that healers make some form of material gain	7
1.3 Mental health credentials	Patients believe healers lack the necessary credentials	11
1.4 Suggestions	Informal Health Providers (IHPs) made suggestions as to how to break these biases	10
2. Cultural misunderstanding and stigma of mental health	General misunderstandings and often negative associations about mental health	37
2.1 Lack of knowledge	No knowledge about mental health	12
2.2 Stigma around mental disorders	Negative beliefs about mental illness	7
2.3 Social support	Partly because of stigma, families and friends do not support the mentally ill	16
2.4 Fear of treatment	Fears about the hospital testing and procedures for mental disorders	5
2.5 Suggestions	IHPs provided opportunities that can be used to reduce these negative perceptions	12
Between screening and treatment		
3. Resource barriers	Lack necessary resources (financial, time, etc.) to travel to hospital for treatment	61
3.1 Patient resources	Patients lack funds to pay for public transport to the hospital, or for food (requiring work at time of appointment)	38
3.2 IHP resource barriers	IHPs lack resources for themselves and for supporting their patients	33
3.3 Suggestions	IHPs suggest ways of dealing with the above barriers	10
4. Resistance to treatment	Patients resist treatment in multiple ways (e.g. illness denial, refusal to go to hospital, substance addiction)	25
4.1 Suggestions	IHPs identify ways of strengthening the willingness of patient referral for those suffering from mental illnesses	11
5. Limitations of the referral system	Lack of recognition on the limit of IHPs’ role in the task-sharing model, resulting in responsibilities outside their role as referrers	25
5.1 Suggestions	IHPs emphasized the need for specialized training such as counselling to know how to motivate patients who have lost hope, to seek further treatment without being accompanied by IHPs	5
During treatment		
6. Treatment infrastructure issues	Many logistical challenges are faced upon arrival to the hospital	39
6.1 Procedural issues	Some untrained hospital staff often do not recognize referral forms	24
6.2 Drug availability	Drugs are often unavailable at the hospital and patients are forced to purchase elsewhere	9
6.3 Limited medical staff	There are often limited medical staff and long queues at the health facilities	6
6.4 Suggestions	IHPs suggested how to improve infrastructure issues	5

### Mistrust of IHPs

The most commonly voiced challenge by IHPs (traditional and faith healers) during the screening phase was mistrust from their patients. The interviewees expressed that often their patients were prejudiced towards IHPs and did not share the same belief systems. Various participants said that their patients seldom associate traditional and faith healers with hospital referrals and question ‘who taught you this?’ and ‘how this person knows this?’ This scepticism explains why these IHPs stated they are ‘not taken seriously’ and believe that their patients ‘refuse to listen or lose confidence in them’, based on the assumption that IHPs have no role in referring them to formal facilities.

A 30 year-old faith healer illustrated the mistrust that emerges between themselves and the patients:

‘….Many people these days have ideologies that many preachers are using evil spirits…and the patient or those around him may think we want to use him to take advantage of him in a bad way. So by the time you reach these people, it becomes a challenge.’

Besides patients’ beliefs about IHPs, participants also said that patients challenge their credentials and motives for referral. Focus group participants from all categories of IHPs stated that they are often forced to ‘explain who [they] are’ because they have ‘no uniform’ to identify them. A 55-year-old female CHW humorously stated that, ‘Even the Chief puts on uniform while on duty (*laughter*). That’s true, right?’ A 30-year-old male faith healer also stated that, ‘…When we are explaining to these people about our training, they challenge us and ask if we were given certificates?’ Moreover, respondents stated that patients often assume that IHPs are taking ‘advantage’ or are acquiring some form of secondary or material gain by referring them to the hospital. As expressed by a 36-year-old traditional healer, ‘[patients] think you have a hidden agenda. They assume you are going to claim money on their behalf’.

In the discussions related to such mistrust, participants offered a number of suggestions to promote recognition. One faith healer in his late forties said, ‘…the county needs to recognize… that we are in this sector’. To counteract patients’ scepticism about the IHPs’ credentials, a 52-year-old female CHW mentioned the importance of having something for identification purposes. Participants in nearly all groups gave very concrete suggestions such as ‘certificates’, ‘letters’, or ‘T-shirts’ that identify them as trained providers. Another IHP recommended the need for extra efforts to convince patients about their new roles as mental health providers. He recommended putting up ‘posters’ and creating ‘awareness in churches, the Sub-chief’s, [and] the chief’s place. In a different but parallel vein, a 77-year-old male traditional healer recommended allotting time to build rapport with patients. He explained that, ‘…we need to bring him [patient] closer and be friends, and after we have become friends with him, there is nothing he can’t tell since he will be free. Therefore, the most important thing is to create the rapport first’. Participants believed that future efforts should include measures to create awareness among the general public and, if patients are still mistrustful, that it would be crucial to incorporate rapport-building time into the referral process.

Participants made an important observation on the positive reaction at health facilities on referral of patients, which was considered to be better than before (prior to training), when their referral notes were disregarded. An 83-year-old male traditional healer explained that ‘now the challenge is that in the past we (herbal doctors) were not taken seriously by modern doctors. For instance, after we referred patients with a referral note, it was torn into pieces but now from last month to date, I have referred five people and they have been received, and the disrespect that was there is no longer there’.

### Cultural misunderstanding and stigma of mental health

A second challenge during the screening phase was cultural misunderstanding and stigma related to mental illness. Participants explained that their patients’ lack of knowledge about mental illness often posed a challenge in working with them, saying that there is ‘ignorance’ about mental illness and that ‘good language’ is needed to effectively communicate with patients. One of the healers, aged 54, said: ‘….This idea of mental problems, people have not been enlightened much.’ The interviewees also said that most often patients have negative and stigmatizing beliefs related to mental illness, which is commonly associated with ‘fear’ and being ‘mishandled’ or ‘bewitched’. A 59-year-old male traditional healer explained that ‘…stigma is a serious problem depending on the way I have seen people think. They think people have madness…’. This stigma resides not only in the individuals, but also in the families and communities in which they live. Numerous participants described the problem of patients’ lack of social support from friends and family regarding their mental health problems and their desire to seek help. One 36-year-old female CHW stated: ‘Sometimes you go meet someone…you find he has a mental disorder, but the people to support him so that he can get to hospital are not there because they see him as someone who is not important and have thus given up.’ A related concern was patients’ and their families’ fears and misunderstanding about hospital testing and procedures related to mental illness. The heightened stigma regarding HIV and AIDS permeates the cultural landscape and creates a sense of fear about other medical procedures. One CHW in her mid-fifties explained that some of them had previously worked with individuals who were diagnosed with HIV and AIDS and described how the shame related to this diagnosis affected the patients’ help-seeking behaviours:

‘…when we were following patients who are infected with HIV/AIDs, they found it difficult to come out/reveal themselves because they didn’t know what to expect, they were shying off and they just wanted to keep their status to themselves even when it was affecting them. This is the same when we are referring them [for mental illness] because as we talk to them, they feel good about it but it becomes a bit difficult for them to actually come out and speak about it.’

Various IHPs suggested potential opportunities to alleviate mental health stigma such as psycho-education among community members and the importance of visiting hospitals. A CHW in her mid-fifties emphasized that this strategy was successful in an earlier activity that educated community members about the benefits of referring women to deliver their baby in a hospital.

### Resource barriers

During the phase between screening by an IHP and hospital treatment, participants described finances and time as a central challenge. Issues primarily related to poverty included the lack of resources among patients (mentioned 38 times) and health providers (mentioned 33 times). Participants said that patients could not pay for transport to the hospital, or lacked food at the time of referral, which required them to earn money rather than seek treatment. For example, a 37-year-old male faith healer explained that ‘people are there who need help but there is a problem. How do they get to the facility? Others need to be given food and transport’. Another male faith healer aged 45 stated that ‘you can meet someone with a mental problem and maybe they have gone for days without food and because he is hungry you find he has no peace or faith in you’.

These financial and resource issues were experienced not only by patients but also by IHPs. Many IHPs faced similar structural challenges, or an additional burden related to their patients’ poverty. Various healers, including a 54-year-old male traditional healer, described a cultural and community expectation for care that extended beyond the IHPs’ goals of referral or treatment, and resulted in a burden on their own families and lives:

‘We cook for the patient when they visit us, we leave our work, we take them to hospital, and where we come from we also have people to take care of, just like everyone else.’

As a solution to such barriers, many IHPs suggested the need to provide transport such as motorbikes or bicycles to transport needy or critical patients to hospital, or refunding the costs related to accompanying patients to hospital. Others also stated that if the IHP suspects that a patient has a mental illness, the referral procedure should be discussed between the patient and the IHP in order to reach a consensus before writing the referral note. This would prevent patients from changing their mind after their meeting with IHPs.

### Resistance to treatment

A second challenge was patients’ resistance to treatment. Patients demonstrated their resistance in a variety of ways including denying their illness, refusing to go to the hospital, or relying on substances instead of the recommended treatment. One CHW aged 42 explained that some patients lacked insight into their mental illness:

‘…you screen someone and you find that the person has dementia, then you give him referral to the hospital but when he gets to the hospital, he refuses that sickness. Particularly, when he is told that he has dementia, he listens to the way it is pronounced then he refuses the illness.’

Another 46-year-old female community health worker described a related scenario:

‘…another one I met after he had agreed and screening him, he said he can’t stop taking alcohol, and says “don’t even give me your referral because you’re trying to see how I can stop taking alcohol, and I can’t stop taking alcohol”.’

One participant compared getting patients to the hospital to ‘a fight’ and was disillusioned to find out that only four of the 30 patients he had referred had gone to the hospital.

All IHPs stated that ways of strengthening patients’ willingness to be referred include creating rapport and respect and providing an atmosphere where the patients can freely express their opinions.

### Limitations of the referral system

Some participants described challenges arising from being taxed with responsibilities beyond their role as referrers. Although participants reported that they believe their responsibility should entail only screening and referral, many said that follow-up procedures such as going with patients to the hospital, remaining with them throughout the duration of their time there, facilitating scheduling of follow-up appointments, and helping the patient understand the medications prescribed often complicated this process because of a lack of recognition on the limits of their role in the task-sharing model. As explained by one 46-year-old female CHW:

‘…To me, we get a challenge especially for those with bipolar because it forces us to go with them to the facility and you find that we spent a lot of time, many hours that we did not plan, because of following them up and knowing how they are taking medications…’

Moreover, participants indicated that some of their patients had ‘lost hope’ and that IHPs needed specialized training in ‘counselling’ to know how to motivate patients to seek further help without necessarily being accompanied by IHPs unless they are unstable.

### Treatment infrastructure issues

Once a patient was receiving hospital treatment, the IHPs described several challenges related to lack of infrastructure. In particular, four related sub-themes emerged: procedural issues (mentioned 24 times), drug availability (nine times), limited medical staff, and related suggestions to address these problems. First, a number of participants described challenges related to procedural issues such as hospital staff, who were not trained because they were outside the scope of study (the task-sharing model involved a sub-set of hospital staff), not recognizing the referral forms. For example, a female traditional healer aged 65 stated that ‘when this patient gets to the hospital and goes to a different person….she is told that the referral note is not known, then she comes back to me with it’.

A second challenge was the lack of drugs, so that patients were required to go elsewhere to purchase the prescribed medications. For example, a 45-year-old male CHW explained that ‘some people are going to the hospital and when they come back they tell us they did not get drugs and we [providers] were not given drugs’.

Sometimes patients were referred and arrived at the hospital, only to find limited medical staff and long waiting times. For example, one respondent said:

‘I refer people from my village together with other IHPs and those people become too many [congested] in the hospital and thus due to the high numbers of referrals, there is workload in the health facilities and this becomes a big problem.’

Several IHPs offered suggestions related to the challenges experienced in treatment infrastructure since some of the patients were unfamiliar with hospital settings due to their reliance on IHPs. For example, a 32-year-old female CHW suggested:

‘I was asking if it could be possible for you to just appoint one CHW to be there because when they are given those referrals they do not know where to start and others have never been to the hospital…’

Another male faith healer, aged 37, felt a change of referral system would help and emphasized the importance of maintaining its status through the multi-stakeholder approach involving building capacity for CHWs, THs and FHs and formal health workers. He suggested the need to train more providers to reach the wider community.

‘…so if the referral system continues to operate this way (nicely) and the organization works hard to train more people the way it trained many of us and we reached many people, and the government supports such organizations, many people will be reached.’

## Discussion

To the best of our knowledge, this is the first study to elucidate the unique challenges faced by IHPs in a task-sharing system in Kenya. These findings demonstrate that although most LMICs embrace the task-sharing model, it is crucial to be aware of challenges likely to hinder implementation as well as opportunities to consider before adopting it.

Previous research documenting the widespread use of IHPs in sub-Saharan Africa [14, 26] has led many to recommend collaborative task-sharing interventions between formal health systems and IHPs [4, 5, 8, 20, 27]. For this study, the interviewed IHPs included CHWs, THs and FHs. Much of the task-sharing literature in LMICs has explored the integration of CHWs into the formal mental health system [28, 29], but none has specifically documented the challenges encountered by THs and FHs [4] or IHPs overall. Therefore, this article sheds further light on the challenges encountered by such IHPs and provides insight, through participants’ suggestions, on ways to address these challenges. With this information, we hope that policy-makers, researchers, and mental health providers can tailor their efforts to create more efficient and sustainable mental health systems.

One of the most commonly described challenges was lack of resources experienced both by IHPs and their patients. Often, individuals seen by IHPs were unable to pay for transport to the healthcare facilities, to commit time away from their work to go to the hospital, and in the worst of cases, feed themselves. The IHPs expressed feeling compelled to give some money to patients in these situations. However, these actions often led to resentment in other encounters because patients expected to receive the same resources, which the IHPs could not afford. In effect, a shortage of resources fundamentally affects the reputation and relationships the IHPs are seeking to establish in communities in Kenya.

In line with these findings, researchers found that the main obstacle to creating an effective task-sharing initiative in LMICs is the lack of resources [9]. Distance and transport costs have also been linked to low acceptability of treatment in rural areas especially where patients have to go to hospitals to receive care [30]. These costs have also been reported outside the task-sharing literature and have been shown to have a negative impact on patients’ uptake of services in various countries such as Kenya [31], Uganda, Liberia, Nepal [32], Bangladesh [33] and Finland [34]. Perhaps in Kenya, a successful task-sharing scale-up model may involve capacity building for most IHPs, who are spread across villages, to promote accessibility, as well as providing emergency transport services such as motorbikes or ambulances for severe cases.

Strengthening capacity building would also need to extend beyond the trained IHPs to all staff based at healthcare facilities rather than a sub-set within them. A variety of treatment infrastructure issues were reported in hospital settings. Participants explained that in some instances they would refer patients to the nearest hospital, only for the patient to be turned away for infrastructure-related reasons, such as some hospital staff not recognizing the referral note. In a study in South Africa where CHWs were trained to refer and coordinate community-based care, Petersen and Pillay [29] found similar issues and demonstrated the crucial need to sensitize all hospital staff about IHPs’ roles and duties in developing a task-sharing initiative. In addition, participants reported that their patients often went to the hospital with the expectation of receiving medical or pharmacological treatment, only to return home untreated. This was as a result of lack of drug availability, patients being unable to pay for the drugs being offered, or too few doctors to attend to the number of patients seeking treatment. These findings corroborate those found in other countries [32, 35, 36].

Similar to literature that explored task-sharing interventions among CHWs [29], a recent study conducted in public hospitals in Kenya showed that people seeking mental health care are unable to pay for essential psychotropic medication at the primary-care clinic [37]. If continuous monitoring for distribution of such medication is established and maintained, and the number of cases detected at the community level translated into demand for medication, this would improve treatment adherence, strengthen the mental health system and reduce the treatment gap related to lack of resources. Continuous supply of drugs forms an essential component of a community-based mental health service provision [38].

Although previous research has shown that IHPs are well-respected and commonly sought for mental health treatment in Kenya [17], in this study the IHPs shared that individuals expressed mistrust towards them. Interestingly, the mistrust was largely related to questioning IHPs’ ability to refer patients’, a role IHPs, particularly THs and FHs, have not historically held [39]. Many participants reported that those suffering from a mental illness believed they lacked the necessary credentials to recognize and understand their problems. This affected IHPs’ ability to refer individuals in need of care. To address this issue, participants recommended that community leaders could promote awareness about mental health work. Community awareness of IHPs’ novel role in mental health care could help reduce misunderstanding and concurrently reduce resistance to treatment which was also reported as a challenge. According to the participants’ reports, a number of patients refused to go to the hospital or denied their illness. By developing more community awareness about IHPs’ role and mental health more generally, patients will likely become more open to hospital referrals.

Interestingly, in comparison to what has been found in previous research [5, 20], health staff received patients referred by IHPs warmly and respectfully. This promising finding suggests that collaboration through task-sharing approaches between informal and formal health providers might reduce mistrust and promote understanding among them. Burns [40] has recommended that IHPs can be integrated successfully into the pathway to care of patients with mental illness and into an adequate community and population-based system that allows identification of possible cases for further intervention.

Participants likewise expressed grievances about the limitations of the referral system, given that they had to perform various responsibilities outside of their designated roles. Patients would at times request that the IHPs manage their logistics, accompany them to procedures, or provide them with individual counselling. IHPs’ confusion about the scope of their role and doubts about how to manage difficulties with patients points to the need for ongoing professional supervision. In former task-sharing initiatives, IHPs have benefitted from professional mental health supervision [9], which has helped them obtain confidence and new skills to overcome challenges [41]. Additionally, mental health experts have stated that supervision should be imperative for task-sharing models, as it ensures sustainability and the provision of adequate care [42]. Supervision could be an excellent means through which to complement the IHPs’ former training, clarify their doubts, and enhance the general effectiveness of the task-sharing referral system.

## Limitations

Our findings are limited to IHPs who had received a task-sharing mental health programme for the first time since this was the first such experience in the region related to mental health. Even though these findings are important, it is crucial to elucidate more opinions after continued supervision and practice.

## Conclusions

Findings from this study illustrate the commitment of different IHPs with limited training to a task-sharing model under difficult situations to increase the access to good quality mental health care for patients in remote settings. The positive uptake of this model by the informal sector, as explained by promotion of trust and respect between formal and informal providers, and minimal attitudinal barriers by the IHPs, is a huge step to reducing the mental health treatment gap. While the resource barriers and lack of psychotropic medicines hinder a task-sharing model in Kenya, these issues are solvable and could be contextually addressed through continuous dialogue, monitoring psychotropic medicine distribution at health facilities, and translating the number of cases detected at the community level to demand. The use of existing resources in the communities by strengthening collaboration between informal and formal health providers through establishing referral pathways underpins this model. Even though the challenges for CHWs, THs and FHs are discussed collectively, it is crucial to note that some challenges are specific to particular providers due to their role before the introduction of a task-sharing model. For instance, mistrust in terms of referral was seen among traditional and faith healers, who are known to treat mental illnesses using various alternative treatments rather than referring to health facilities. As such, patients start losing confidence in the IHPs in general. However, opportunities specific to these challenges were identified during the discussions.

Investment in capacity building with supportive supervision for IHPs also needs to be established in regions that have inadequate health personnel. The recently launched mental health policy [43] in Kenya incorporates integration of IHPs in mental health care in collaboration with the ministry on health matters to ensure adequate trained mental health workforce; promises to establish partnerships between public, private and voluntary sectors; aims to strengthen the institutional and procurement systems linkage between Kenya Medical Supplies Agency (KEMSA) and institutions providing psychotropic drugs to users; and finally, works towards adopting frameworks to ensure improved access to essential psychotropic medicines.

It is worth noting that attitudinal barriers such as beliefs about mental illness are less reported in this study compared to structural barriers such as lack of time, resources and medicines. Previous studies have revealed that attitudinal factors are more frequently identified as barriers to seeking help than structural factors [44]. The assumption is that introduction of the task-sharing model could partly contribute to the reduction of attitudinal barriers, although this cannot conclusively be deduced. There would be a need for more studies that would require patients and key informant views as well as quantitative aspects to ascertain whether this is the case.

Finally, the expansion of this type of research has promising implications as it focuses on using individuals from rural communities to facilitate access to mental health care. Future studies may focus on including more providers in the same and other regions in Kenya, and LMICs more widely, and offer more suggestions on how to improve the current mental health system including conducting home visits to reduce transport barriers to health facilities.

## Abbreviations

LMICs: low- and middle-income countries
IHPs: informal health providers
CHWs: community health workers
FHs: faith healers
THs: traditional healers
FGDs: focus group discussions
MUERC: Maseno University Ethics Review Committee
